# Data and programs in support of network analysis of genes and their association with diseases

**DOI:** 10.1016/j.dib.2016.07.022

**Published:** 2016-07-19

**Authors:** Panagiota I. Kontou, Athanasia Pavlopoulou, Niki L. Dimou, Georgios A. Pavlopoulos, Pantelis G. Bagos

**Affiliations:** a Department of Computer Science and Biomedical Informatics, University of Thessaly, Papasiopoulou 2-4, Lamia 35100, Greece; b Lawrence Berkeley Lab, Joint Genome Institute, United States Department of Energy, 2800 Mitchell Drive, Walnut Creek, CA 94598, USA

**Keywords:** Gene-disease associations, Gene-gene networks, Disease-disease networks

## Abstract

The network-based approaches that were employed in order to depict the relationships between human genetic diseases and their associated genes are described. Towards this direction, monopartite disease-disease and gene-gene networks were constructed from bipartite gene-disease association networks. The latter were created by collecting and integrating data from three diverse resources, each one with different content, covering from rare monogenic disorders to common complex diseases. Moreover, topological and clustering graph analyses were performed. The methodology and the programs presented in this article are related to the research article entitled “*Network analysis of genes and their association with diseases*” [1].

**Specifications Table**

**Table:** 

Subject area	Systems biology
More specific subject area	Gene-disease networks
Type of data	Figure, text files, Cytoscape Network file
How data were acquired	Data were acquired from the publicly available databases: OMIM, GAD, GWAS, UniProtKB, ICD, HGNC
Data format	Processed, analyzed
Experimental factors	Gene-disease association data were analyzed using Perl and R scripts and Cytoscape.
Experimental features	Gene-gene and disease-disease networks were constructed.
Data source location	Department of Computer Science and Biomedical Informatics, University of Thessaly, Lamia, Greece
Data accessibility	Data are provided with this article.

**Value of the data**

• The need for integrating complementary data from different sources to biological networks is further highlighted in this study.
• Important, previously unknown, associations between genes and diseases were revealed.
• Based on the constructed disease-disease networks, diseases with apparently distinct phenotypic manifestations were found to share a common genetic background. This finding could be utilized in network pharmacology.

## 1 Data

**Fig.1:**
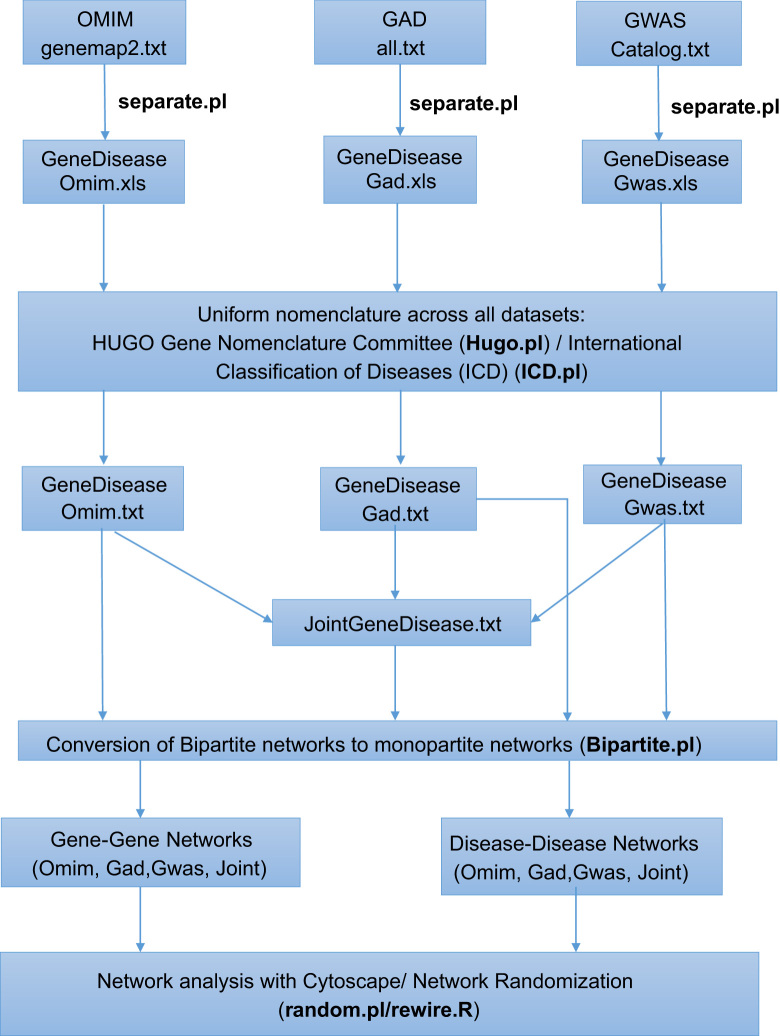
Flow Diagram of the data analysis.

The overall procedure of the data analysis is shown illustratively in Fig. 1. The Perl (Supplementary Files 1-5) and R (Supplementary File 6) programs used for data analysis are indicated. A complete description of the data and methodology is presented in [1].

## 2 Experimental design, materials and methods

### 2.1 Data collection

Disease-gene association data were collected and integrated from three diverse publicly available, comprehensive resources (NCBI׳s OMIM [2], NIH׳s GAD [3] and NHRI GWAS Catalog [4]). As a given disease can be associated with more than one gene, a script was written in Perl to separate the multiple entries (Supplementary File 1; separate.pl).

### 2.2 Disease and gene nomenclature

In order to maintain a consistent nomenclature and classification for diseases in our analysis, the naming conventions described in the International Classification of Diseases (ICD) were used. The disease terms from the three databases were converted to ICD terms with the use of a Perl script (Supplementary File 2; ICD.pl). Moreover, in order to maintain a uniform nomenclature across all datasets, all genes from our three databases along with the ones from UniProtKB [5] were converted to the official HGNC (HUGO Gene Nomenclature Committee) [6] gene symbols using a Perl script (Supplementary File 3; Hugo.pl).

### 2.3 Network processing and analysis

The bipartite networks of gene-disease associations were converted to monopartite networks of gene-gene and disease-disease interactions, by using a Perl script (Supplementary File 4; Bipartite.pl). This functionality is not available in other network analysis packages and we incorporated it in a publicly available web-server, PowerClust, which is available at: http://www.compgen.org/tools/powerclust. PowerClust, is an easy-to-use web application for clustering analysis, network processing and visualization. Moreover, randomization procedures were performed in order to determine whether the highly connected nodes in the original networks have a degree that cannot occur simply by chance given the other properties of the networks (Supplementary File 5; Random.pl). Finally, the robustness of the topological features of the projected gene-gene and disease-disease networks was assessed by employing a bipartite-specific rewiring algorithm [7] to test whether the degree distributions of the projected monopartite networks are kept stable in the randomized gene-gene/disease-disease networks compared to the initial ones (Supplementary File 6; Rewire.R). The JOINT gene-disease network (generated by combing data from the individual databases) is provided as a cytoscape network file.

## Appendix A Supporting information

**Supplementary File 1.** A Perl script which separates the multiple entries between gene- disease associations (“separate.pl”). **Supplementary File 2.** A Perl script which converts the disease terms from the three databases to ICD terms (“ICD.pl”). **Supplementary File 3.** A Perl script which converts the gene terms from the three databases to the official HGNC (HUGO Gene Nomenclature Committee) gene symbols (“Hugo.pl”). **Supplementary File 4.** A Perl script which converts the bipartite networks to monopartite networks(“Bipartite.pl”). **Supplementary File 5.** A Randomization method implemented in Perl (“Random.pl”). **Supplementary File 6.** A bipartite-specific rewiring algorithm implemented in R (“Rewire.R”).
