# Supplementary material for: Data and programs in support of network analysis of genes and their association with diseases
Source: Data Brief. 2016 Jul 19;8:1036–9. doi: 10.1016/j.dib.2016.07.022 (PMC4969244; doi:10.1016/j.dib.2016.07.022)
Supplement: Supplementary material [file mmc1.doc]

**Conflict of Interest**

The authors declare that they do not have conflicts of interest. The funding source of the study is acknowledged in the respective section.
